# Nutritional and bioactive constituents and scavenging capacity of radicals in *Amaranthus hypochondriacus*

**DOI:** 10.1038/s41598-020-71714-3

**Published:** 2020-11-17

**Authors:** Umakanta Sarker, Shinya Oba

**Affiliations:** 1 Department of Genetics and Plant Breeding, Faculty of Agriculture, Bangabandhu Sheikh Mujibur Rahman Agricultural University, Gazipur, 1706 Bangladesh; 2 Laboratory of Field Science, Faculty of Applied Biological Sciences, Gifu University, Yanagido 1-1, Gifu, Japan

**Keywords:** Biochemistry, Natural variation in plants

## Abstract

*A. hypochondriacus* leaves contained ample phytopigments including betalain, anthocyanin, β-xanthin, β-cyanin, and bioactive phytochemicals of interest in the industry of food. We have been evaluating the possibility of utilizing phytopigments of amaranth and bioactive constituents for making drinks. Therefore, we evaluated bioactive phytopigments and compounds including the potentiality of antioxidants in *A. hypochondriacus* leaves. *A. hypochondriacus* leaves have abundant protein, carbohydrates, and dietary fiber. We found considerable levels of inorganic minerals including magnesium, calcium, potassium (3.88, 3.01, 8.56 mg g^−1^), zinc, manganese, copper, iron (16.23, 15.51, 2.26, 20.57 µg g^−1^), chlorophyll *b*, chlorophyll *ab* chlorophyll *a* (271.08, 905.21, 636.87 μg g^−1^), scavenging capacity of radicals (DPPH, ABTS^+^) (33.46, 62.92 TEAC μg g^−1^ DW), total polyphenols (29.34 GAE μg g^−1^ FW), β-xanthin, betalain, β-cyanin (584.71, 1,121.93, 537.21 ng g^−1^), total flavonoids (170.97 RE μg g^−1^ DW), vitamin C, β-carotene, carotenoids (184.77, 82.34, 105.08 mg 100 g^−1^) in *A. hypochondriacus* leaves. The genotypes AHC6, AHC4, AHC11, AHC5, and AHC10 had a good scavenging capacity of radicals. Polyphenols, phytopigments, flavonoids, and β-carotene of *A. hypochondriacus* had potential antioxidant activity. Extracted juice of *A. hypochondriacus* can be an ample source of phytopigments and compounds for detoxification of reactive oxygen species (ROS) and attaining nutritional and antioxidant sufficiency.

## Introduction

In the globe, 795 million people are affected by a continuous deficit in calories for the scarcity of sufficient foods^1^. Around 2 billion people are affected by the deficiency of vitamins or minerals^2^. The main source of energy in the human diet is the consumption of staple foods regularly even though these are deficient in micronutrients, such as iodine, iron, zinc, pro-vitamin A, vitamin E, vitamin C, and carotenoids^3^. As a result, continuous consumption of staple foods in the human diet, consequently resulting in hidden hunger^2^. Hence, we can ensure a balanced and healthy diet by consuming vegetables and fruits as the occurrence of minerals and vitamins along with staple foods. Furthermore, we protect our health and reduce the risk of cancer, cardiovascular disease, and many chronic diseases by feeding fruits and vegetables. Bioactive constituents, such as nutrients, phenolics, pigments, flavonoids, and vitamins contribute to many health benefits^4–6^.

*Amaranthus* genus has a C_4_ photosynthetic pathway that is distributed widely in America, Africa, Australia, Asia, and Europe. Among the seventy species, seventeen are cultivated as edible leafy vegetables and three are cultivated as grain amaranths^7^. *Amaranthus* contains thirteen times more ascorbic acid, twenty times more calcium, eighteen times more pro-vitamin A, and seven times more Fe in comparison with lettuce^8^. It contains protein with essential amino acids including methionine and lysine, carotenoids, dietary fiber, vitamin C, minerals^9–15^. It has abundant phytopigments including betalain, anthocyanin, β-cyanin, carotenoids, β-xanthin, and chlorophylls^16,17^, bioactive compounds including flavonoids, vitamin C, phenolic acids, carotenes^18^. The phytopigments and bioactive constituents quenching ROS and had a magnificent contribution to the industry of food^19–21^. Amaranth is a unique source of betalain, β-xanthin, and β-cyanin. Amaranths have multipurpose uses and widely acclimated vegetables to different abiotic stresses including drought^22–25^ and salinity^26–28^.

The underutilized leafy vegetables, *A. hypochondriacus* originates from North America, possibly by the hybridization of wild *A. powellii* (North American) and *A. cruentus* (cultivated)^29^. Now, *A. hypochondriacus* is extensively cultivated throughout the world including tropical, subtropical and temperate climates. It is used as vegetables, grains, and ornamental plants. It has great diversity and phenotypic plasticity^30^. Mild flavored juvenile leaves and edible fleshy stems of *A. hypochondriacus* are popularly used as leafy vegetables in India, Bangladesh, Asia, and Africa due to nutritive values and taste. *A. hypochondriacus* contains tannin and has astringent flavor. Traditionally, it is used internally for the treatment of diarrhea, excessive menstruation, gargle to soothe inflammation of the pharynx, enhance the healing of ulcerated mouths and externally for nosebleeds and wounds. Yellow, red, and green natural pigments can be used as colorants in foods and medicines^29^.

Currently, researchers and consumers are very much interested in natural antioxidants of vegetables. Natural antioxidants of amaranth include phytopigments (carotenoids, chlorophyll, betaxanthin, and betacyanin), phenolics, ascorbic acids, and flavonoids^19,20^. These natural bioactive constituents protect us from neurodegenerative diseases, cancer, cardiovascular diseases, atherosclerosis, cataracts, emphysema, arthritis, and retinopathy^5,31,32^. These products containing antioxidant properties have a substantial interest to consumers. Many medicinal plants containing phenolics, vitamins C, carotenoids, flavonoids and other non-nutrient constituents are potentially used as an antioxidant with a protective capacity^33^.

Recently, we have been evaluating the possibility of utilizing phytopigments of amaranth containing abundant natural β-xanthin, betalain, β-cyanin, and bioactive compounds of interest in the industry of food^16,17^. It is the first attempt to evaluate nutrients, bioactive components and scavenging capacity of radicals in *A. hypochondriacus*. Hence, in the current investigation was carried out to evaluate the occurrence of bioactive constituents**,** and scavenging capacity of radicals of *A. hypochondriacus* in detail and study the possibility of *A. hypochondriacus* genotypes for making drinks containing high antioxidant constituents and antioxidant activity.

## Results and discussion

### Proximate compositions

Table 1 represents the proximate compositions of *A. hypochondriacus* genotypes. The genotype AHC11 (885.75 g kg^−1^ FW) exhibited the highest content of moisture, whereas the lowest moisture content was obtained from AHC4 (817.85 g kg^−1^ FW). The content of moisture ranged from 817.85 to 885.75 g kg^−1^ FW. As lower moisture contents ensured higher dry matter, the genotype AHC4, AHC8, AHC1, and AHC7 had 16–19% dry matter could be selected for dry matter contents. The content of moisture of *A. hypochondriacus* leaves directly associated with the maturity of the plant. Our obtained results were corroborated with the findings of sweet potato leaves^34^. The remarkable variations in protein content were noticed in *A. hypochondriacus* leaves. The highest protein content was noticed in the genotype AHC2 (61.62 g kg^−1^) followed by that of AHC10, AHC1, AHC6, and AHC11, whereas the lowest protein content was recorded in the genotype AHC8 (47.41 g kg^−1^). Across all genotypes, five genotypes exhibited better protein content over their average value. The genotypes AHC2, AHC10, AHC1, AHC6, and AHC11 had relatively high protein contents for a leafy vegetable. *A. hypochondriacus* provides important roles as a protein source for vegetarians and many people in low-income countries. *A. hypochondriacus* (54.18 g kg^−1^) had much higher protein content in comparison with *A. tricolor* of our earlier study^12^. AHC2 and AHC7 exhibited the highest fat content (4.44, 4.27 g kg^−1^ FW) showing the order of AHC2 = AHC7 ˃ AHC10 ˃ AHC5. The genotype AHC3 exhibited the lowest fat content (2.43 g kg^−1^ FW) which was statistically similar to AHC6, AHC8, AHC1, AHC9, AHC4, and AHC11 with an average of 3.09 g kg^−1^ FW. The fat content in the current investigation agreed to the fat content of sweet potato leaves by Sun et al.^34^. It is known that fat influences the cell function, covering the organs and upholding the temperature of the body. Fats play a vital role in digestion, absorption, and transport of vitamins A, E, K, and D that are soluble in fats.

**Table 1:** The composition of proximate (g kg^−1^ fresh weight) and dietary fiber (g 100 g^−1^ FW) of 11 *A. hypochondriacus* genotypes.

Genotypes	Moisture (g kg^−1^)	Protein (g kg^−1^)	Fat (g kg^−1^)	Carbohydrates (g kg^−1^)	Energy (kcal)	Ash (g kg^−1^)	Dietary fiber (g 100 g^−1^ FW)
AHC1	838.52 ± 6.67 g	59.93 ± 2.19b	2.78 ± 0.03c	50.52 ± 1.15e	56.61 ± 1.56b	48.25 ± 0.87d	8.83 ± 0.87c
AHC2	848.57 ± 2.86f	61.62 ± 1.85a	4.44 ± 0.02a	38.82 ± 1.28h	42.35 ± 2.15e	46.55 ± 0.98e	10.27 ± 0.22a
AHC3	875.24 ± 3.88b	52.52 ± 1.12d	2.43 ± 0.05c	42.96 ± 1.83g	38.15 ± 2.25f	26.85 ± 1.11g	9.33 ± 0.66b
AHC4	817.85 ± 5.42i	52.23 ± 1.68d	2.96 ± 0.02c	70.74 ± 1.26a	58.47 ± 2.35a	56.22 ± 0.88a	9.61 ± 0.27b
AHC5	866.76 ± 5.32d	50.62 ± 1.22e	3.19 ± 0.04b	42.97 ± 1.36g	37.26 ± 2.28f	36.46 ± 1.18f	8.34 ± 0.63c
AHC6	865.66 ± 4.34d	55.12 ± 0.96c	2.65 ± 0.03c	48.01 ± 1.75f	48.26 ± 2.35c	28.56 ± 1.22g	10.03 ± 0.35a
AHC7	836.52 ± 3.75g	51.26 ± 0.78d	4.27 ± 0.02a	62.12 ± 1.62c	46.18 ± 1.29c	45.83 ± 0.87e	7.95 ± 0.38d
AHC8	826.54 ± 5.62h	47.41 ± 1.14f	2.38 ± 0.02c	69.12 ± 1.72b	55.96 ± 2.28b	54.55 ± 1.13c	7.87 ± 0.66d
AHC9	853.58 ± 5.48e	50.10 ± 1.52e	2.78 ± 0.01c	57.10 ± 1.18d	46.75 ± 2.19c	36.44 ± 1.18f	8.27 ± 0.38c
AHC10	867.71 ± 4.48c	59.77 ± 1.13b	3.65 ± 0.04b	31.59 ± 1.17i	44.25 ± 2.23d	37.28 ± 1.14f	8.23 ± 0.33c
AHC11	885.75 ± 4.85a	55.45 ± 0.94c	2.48 ± 0.04c	1.95 ± 1.35j	28.95 ± 2.74g	54.37 ± 1.12b	7.26 ± 0.15d
Mean	867.71	54.18	3.09	46.90	45.74	42.85	8.73
Significance	**	**	**	**	**	**	**
CV%	2.72	1.51	0.26	0.74	0.76	0.54	0.32

*CV* coefficient of variation; n = 3; **Significant at 1% level.

The highest carbohydrates contents (70.74 g kg^−1^ FW) were recorded in AHC4 followed by that of AHC8 and AHC7, whereas AHC11 exhibited the lowest carbohydrates contents (1.95 g kg^−1^ FW) including an average of 46.90 g kg^−1^ FW. The highest energy (58.47 kcal 100 g^−1^) was found in genotype AHC4, followed by that of AHC8, AHC1, AHC6, and AHC7, while the genotype AHC5 and AHC3 (37.26, 38.15 kcal 100 g^−1^) showed the lowest energy including an average of 45.74 kcal 100 g^−1^ FW. AHC4 (56.22 g kg^−1^ FW) exhibited the highest ash content followed by that of AHC11, AHC8, and AHC1, while AHC9, AHC5, and AT17 had the lowest ash content (36.44, 36.46, 37.28 g kg^−1^ FW, respectively) including a mean value of 42.85 g kg^−1^ FW.

The remarkable variations were noticed in the content of the digestive fiber of 11 *A. hypochondriacus* studied. The highest dietary fiber was noticed in AHC2 and AHC6 (10.27, and 10.03 g 100 g^−1^) followed by AHC4, while AHC11 had the lowest dietary fiber contents (7.26 g 100 g^−1^) which was statistically similar to the genotype AHC8 and AHC7 with an average of 8.73 g 100 g^−1^. For mitigation of palatability, food digestibility, and remedy of constipation, dietary fiber has a crucial role^9^. In this study; we observed that *A. hypochondriacus* has abundant protein, carbohydrates, dietary fiber, and moisture. The moisture contents observed in *A. hypochondriacus* leaves was greater than our previous studies of red morph amaranth^35^, weedy amaranth^36^, green morph amaranth^37^, stem amaranth^38^, while the moisture contents of *A. hypochondriacus* leaves were corroborated with our previous studies of *A. blitum*^39^. In contrast, the protein contents observed in *A. hypochondriacus* leaves were greater than our previous studies of red morph amaranth^35^, *A. viridis* weedy amaranth^36^, green morph amaranth^37^, stem amaranth^38^, and *A. blitum*^39^, while the protein contents of *A. hypochondriacus* leaves were corroborated with our previous studies of *A. spinosus* weedy amaranth^36^. However, The carbohydrates contents observed in *A. hypochondriacus* leaves were greater than our previous studies of *A. spinosus* weedy amaranth^36^, while the carbohydrates contents of *A. hypochondriacus* leaves were lower than red morph amaranth^35^, green morph amaranth^37^, *A. viridis* weedy amaranth^36^, stem amaranth^38^, and *A. blitum*^39^. The digestible fiber obtained from *A. hypochondriacus* leaves were greater than our previous studies of red morph amaranth^35^, green morph amaranth^37^, stem amaranth^38^, and *A. blitum*^39^, while digestible fiber obtained from *A. hypochondriacus* leaves were lower than our previous studies of weedy amaranth^36^.

### Macro and microelements compositions

The macro and microelements composition of *A. hypochondriacus* is presented in Table 2. In this study, the K content ranged from 5.19 mg g^−1^ to 8.56 mg g^−1^ FW. The lowest K content was recorded in the genotypes AHC3, AHC6, AHC1, AHC9, AHC4, AHC11, AHC7, AHC2, and AHC10, while the highest K content was exhibited in the genotype AHC8 followed by that of AHC5, with a grand mean value of 5.74 mg g^−1^ FW. Two genotypes had higher K contents than the mean K content. The highest Ca content was noted in the genotype AHC8 (3.01 mg g^−1^), followed by that of AHC2, whereas, the lowest Ca content was reported in the genotypes AHC4 (1.89 mg g^−1^) with an average of 2.43 mg g^−1^ FW. Six accessions exhibited high Ca contents than the corresponding mean. The highest magnesium content was recorded in the genotype AHC8 (3.88 mg g^−1^) and the lowest magnesium content was observed in AHC4 (3.20 mg g^−1^), including an average of 3.41 mg g^−1^ FW. In the current investigation, Mg content had not varied considerably among the genotypes (3.20 to 3.88 mg g^−1^ FW). Our results showed that *A. hypochondriacus* genotypes (FW basis) had remarkable Ca (3.01 mg g^−1^), K (8.56 mg g^−1^), and Mg (3.88 mg g^−1^) which was more pronounced than Mg, Ca, and K content of different amaranth species including *A. hypochondriacus* of Jimenez-Aguiar and Grusak^40^. Jimenez-Aguir and Grusak^40^ observed that different amaranth species including *A. hypochondriacus* had abundant Mg, Ca, and K in comparison with spider flower, kale, black nightshade, and spinach. K content of *A. hypochondriacus* leaves was greater than the K content of our previous studies of green morph amaranth^37^, while K content obtained from *A. hypochondriacus* leaves was lower than the K content of our previous studies of weedy amaranth^36^. Ca content observed in the current investigation were corroborated with green morph amaranth^37^ and weedy amaranth^36^. Mg obtained from *A. hypochondriacus* leaves were greater than the Mg content of our previous studies of green morph amaranth^37^ and *A. spinosus*^36^, while Mg obtained from *A. hypochondriacus* leaves were corroborated with the Mg content of our previous studies of *A. viridis*^36^.

**Table 2:** The composition of minerals (Macro mg g^−1^ FW and micro µg g^−1^ FW elements) of 11 *A. hypochondriacus* genotypes.

Genotypes	Macroelements (mg g^−1^ FW)			Microelements (µg g^−1^ FW)			
	K	Ca	Mg	Fe	Mn	Cu	Zn
AHC1	5.19 ± 0.13c	2.69 ± 0.12c	3.38 ± 0.15c	10.60 ± 0.72f	11.86 ± 0.16d	1.10 ± 0.04e	9.32 ± 0.21e
AHC2	5.21 ± 0.12c	2.94 ± 0.10b	3.44 ± 0.18c	11.76 ± 0.42e	7.72 ± 0.12f	1.17 ± 0.02e	7.25 ± 0.15f
AHC3	5.79 ± 0.12c	3.01 ± 0.24a	3.31 ± 0.12c	10.49 ± 0.62f	12.69 ± 0.13c	1.61 ± 0.02c	13.88 ± 0.15c
AHC4	5.30 ± 0.10c	1.89 ± 0.16f	3.20 ± 0.13c	10.98 ± 0.23f	7.66 ± 0.17f	1.01 ± 0.02e	7.25 ± 0.15f
AHC5	6.64 ± 0.21b	2.14 ± 0.11e	3.58 ± 0.12b	17.28 ± 0.45b	14.51 ± 0.18b	2.11 ± 0.03a	16.23 ± 0.14a
AHC6	5.48 ± 0.14c	2.28 ± 0.15e	3.38 ± 0.08c	14.32 ± 0.48d	6.84 ± 0.14g	1.11 ± 0.01e	7.45 ± 0.16f
AHC7	5.21 ± 0.15c	2.13 ± 0.12e	3.31 ± 0.08c	7.48 ± 0.55g	7.59 ± 0.16f	0.90 ± 0.06f	9.01 ± 0.16e
AHC8	8.56 ± 0.15a	2.45 ± 0.17d	3.88 ± 0.10a	15.25 ± 0.22c	15.51 ± 0.10a	1.36 ± 0.02d	15.68 ± 0.17b
AHC9	5.19 ± 0.07c	2.62 ± 0.13c	3.35 ± 0.11c	10.35 ± 0.19f	8.54 ± 0.15e	2.26 ± 0.03a	11.51 ± 0.13d
AHC10	5.32 ± 0.08c	2.62 ± 0.15c	3.38 ± 0.16c	20.57 ± 0.48a	5.77 ± 0.12h	1.91 ± 0.05b	6.40 ± 0.12g
AHC11	5.19 ± 0.11c	2.39 ± 0.15d	3.25 ± 0.07c	11.18 ± 0.26e	6.64 ± 0.13g	1.26 ± 0.05d	6.94 ± 0.13g
Mean	5.74	2.43	3.41	12.75	9.58	1.38	9.98
Significance	**	**	**	**	**	**	**
CV%	0.25	0.27	0.14	0.31	0.42	0.13	0.26

*CV* coefficient of variation, *K* potassium, *Ca* calcium, *Mg* magnesium, *Fe* iron, *Mn* manganese, *Cu* copper, *Zn* zinc, n = 3; **Significant at 1% level.

The iron content of *A tricolor* had the significant and pronounced variations regarding genotypes (7.48 µg g^−1^ FW in AHC7 to 20.57 µg g^−1^ FW in AHC10). High iron content was recorded in the genotypes AHC10, AHC5, AHC8, and AHC6. On the contrary, the lowest content of iron was recorded in the genotype AHC3, AHC1, AHC9, and AHC4, including an average of 12.75 µg g^−1^ FW. Four accessions exhibited higher iron contents than their corresponding average value. The highest manganese content was noted in the genotype AHC8 (15.51 µg g^−1^ FW), while the lowest manganese content was noted in the genotype AHC10 (5.77 µg g^−1^ FW) with an average of 9.58 µg g^−1^ FW. The genotypes AHC5, AHC8, AHC3, and AHC1 had high manganese content. The genotypes differed remarkably in copper content (0.90–2.26 µg g^−1^ FW). The highest copper content was noted in AHC9 and AHC5 (2.26 and 2.11 µg g^−1^ FW) followed by AHC10. Four accessions exhibited greater Cu contents than the mean value. The significant and remarkable variations were noted in the zinc content of the studied genotypes (6.40 µg g^−1^ FW (AHC10) to 16.23 µg g^−1^ FW (AHC5). Four genotypes showed better zinc contents than the corresponding mean (9.98 µg g^−1^ FW). Iron and zinc content of *A. hypochondriacus* was greater than the cassava leaves^41^ and beach pea^42^. We observed considerable Mn (15.51 µg g^−1^), Fe (20.57 µg g^−1^), Zn (16.23 µg g^−1^), and Cu (2.26 µg g^−1^) in *A. hypochondriacus* (fresh weight basis). Similarly, Jimenez-Aguiar and Grusak^40^ noted abundant Cu, Fe, Zn, and Mn (FW basis) in various amaranth species with *A. hypochondriacus*. They reported higher Cu, Fe, Mn, and Zn in different amaranth species including *A. hypochondriacus* than spider flower, spinach, black nightshade, and kale. Mn content noticed in the current investigation was much lower than our previous studies of green morph amaranth^37^ and *A. spinosus* weedy amaranth^36^, while it corroborated with our previous findings of *A. viridis* weedy amaranth^36^. Fe content observed in the current investigation were much greater than our previous studies of green morph amaranth^37^ and lower than our previous studies of weedy amaranth^36^. Cu and Zn contents observed in the current investigation was corroborated with our previous studies of green morph amaranth^37^, while Cu and Zn contents obtained from *A. hypochondriacus* leaves were lower than our previous studies of weedy amaranth^36^.

### Composition of phytopigments

Table 3 represents the composition of phytopigments of *A. hypochondriacus* genotypes studied. The chlorophyll *a* content (131.07–636.87 μg g^−1^ FW) exhibited prominent variations among genotypes. The highest content of chlorophyll *a* (636.87 μg g^−1^ FW) was noted in the genotype AHC4, while the genotype AHC9 exhibited the lowest content of chlorophyll *a* (131.07 μg g^−1^ FW). The high content of chlorophyll *a* was noted in the genotypes AHC2 and AHC11. Six genotypes had a higher content of chlorophyll *a* than the mean value. Similar to chlorophyll *a*, chlorophyll *b* content also had significant and progressive variations among 11 *A. hypochondriacus* genotypes studied (71.27–271.08 μg g^−1^ FW). The highest chlorophyll *b* content (271.08 μg g^−1^ FW) was recorded in AHC7, followed by that of AHC4 and AHC11. Conversely, the lowest chlorophyll *b* content was noticed in the genotype AHC9 (71.27 μg g^−1^ FW). The content of chlorophyll *ab* showed significant and remarkable variations (193.98 to 905.21 μg g^−1^ FW). The genotypes AHC4, AHC11, AHC5, and AHC6 exhibited high chlorophyll *ab* content, whereas, the genotype AHC8 exhibited the lowest chlorophyll *ab* content (193.98 μg g^−1^ FW). Six accessions had greater chlorophyll *ab* contents than the mean value. Our studied *A. hypochondriacus* genotypes had considerable chlorophyll *ab* (905.21 μg g^−1^ FW), chlorophyll *a* (636.87 μg g^−1^ FW), and chlorophyll *b* (271.08 μg g^−1^ FW) contents which were greater than the content of chlorophylls of red and green amaranth of Khanam and Oba^43^*.* Chlorophyll *a*, chlorophyll *ab*, and chlorophyll *b* content in the present study were much greater than chlorophyll *a*, chlorophyll *ab*, and chlorophyll *b* content of green morph amaranth^37^, red morph amaranth^35^, stem amaranth^38^, and weedy amaranth^36^ of our earlier study, while these photosynthetic pigments content of this study were lower corroborated with our earlier studies of *A. blitum*^39^.

**Table 3:** Mean performance for phytopigments of 11 *A. hypochondriacus* genotypes.

Genotypes	chlorophyll *a* (μg g^−1^ FW)	Chlorophyll *b* (μg g^−1^ FW)	Chlorophyll *ab* (μg g^−1^ FW)	β-cyanin (ng g^−1^ FW)	β-xanthin (ng g^−1^ FW)	Betalain (ng g^−1^ FW)	Carotenoids (mg 100g^−1^ FW)
AHC1	205.99 ± 3.24i	222.16 ± 0.69f.	428.15 ± 2.16h	385.52 ± 1.26g	372.19 ± 0.78g	757.71 ± 1.69g	88.29 ± 0.46c
AHC2	523.21 ± 4.24b	178.82 ± 0.99h	702.03 ± 2.24d	302.17 ± 1.38i	308.31 ± 0.75i	610.48 ± 1.79i	96.37 ± 0.34b
AHC3	346.84 ± 2.17g	158.54 ± 0.86i	505.38 ± 1.12g	391.53 ± 1.72f	398.09 ± 0.81f	789.62 ± 1.72f	83.89 ± 0.26d
AHC4	636.87 ± 2.14a	268.34 ± 0.77b	905.21 ± 3.21a	407.94 ± 1.53e	427.55 ± 0.63e	835.49 ± 1.84e	32.77 ± 0.65j
AHC5	504.56 ± 3.34d	231.86 ± 0.78d	736.41 ± 3.18c	484.77 ± 1.32c	492.99 ± 0.88c	977.76 ± 1.87c	82.89 ± 0.48e
AHC6	304.82 ± 3.12h	226.20 ± 0.78e	531.05 ± 2.34f	537.21 ± 1.64a	584.71 ± 0.95a	1,121.93 ± 1.84a	55.38 ± 0.27i
AHC7	432.74 ± 2.07f	271.08 ± 0.67a	703.81 ± 2.23d	343.99 ± 1.55h	346.18 ± 0.97h	690.17 ± 2.36h	59.81 ± 0.29h
AHC8	131.56 ± 2.11j	62.42 ± 0.88k	193.98 ± 3.15j	185.52 ± 1.85k	181.90 ± 0.84k	367.42 ± 2.78k	68.84 ± 0.36f
AHC9	131.07 ± 4.17k	71.27 ± 0.99j	202.33 ± 2.18i	233.87 ± 1.32j	230.57 ± 0.84j	464.44 ± 2.78j	65.91 ± 0.47g
AHC10	441.60 ± 2.08e	217.78 ± 0.67g	659.38 ± 3.18e	453.59 ± 1.58d	467.36 ± 0.92d	920.95 ± 2.45d	105.08 ± 0.76a
AHC11	517.16 ± 1.11c	252.05 ± 0.68c	769.20 ± 1.25b	500.40 ± 1.81b	502.79 ± 0.71b	1,003.19 ± 1.54b	65.55 ± 0.37g
Mean	379.67	196.41	577.10	384.23	392.06	709.68	73.16
Significance	**	**	**	**	**	**	**
CV%	3.67	1.34	1.56	2.24	2.32	1.52	2.33

*CV* coefficient of variation; n = 3; **Significant at 1% level.

The highest content of β-cyanin was noted in the genotype AHC6 (537.21 ng g^−1^ FW) followed by that of AHC11 and AHC5, with an average value of 384.23 ng g^−1^ FW. On the contrary, the lowest β-cyanin content was observed in the genotype AHC9 (233.87 ng g^−1^ FW). The β-xanthin content had significant and considerable variations regarding genotypes which ranged from 181.90 to 584.71 ng g^−1^ FW. The genotype AHC6 exerted the highest β-xanthin content (584.71 ng g^−1^ FW) and the genotypes AHC11, AHC5, and AHC10 had high β-xanthin content. In contrast, the lowest β-xanthin content was recorded in the genotype AHC8 (181.90 ng g^−1^ FW). Six accessions exerted greater β-xanthin contents than the mean value. There were pronounced variations in the betalain content of the genotypes studied. The highest betalain content was observed in the genotype AHC6 (1,121.93 ng g^−1^ FW) and ranged from 367.42 to 1,121.93 ng g^−1^ FW. The high betalain content was noted in the genotype AHC11, AHC5, and AHC10. Whereas, the lowest content of betalain was recorded in the genotype AHC8 (367.42 ng g^−1^ FW). Seven accessions exerted greater betalain contents than the mean value. The highest carotenoid content was found in the genotype AHC10 (105.08 mg 100 g^−1^) and ranged from 32.77 mg 100 g^−1^ to 105.08 mg 100 g^−1^. The high carotenoids were recorded in the genotype AHC2, AHC1, and AHC3. Five accessions exerted greater carotenoids than the mean value. The considerable β-xanthin (584.71 ng g^−1^ FW), chlorophyll *a* (636.87 μg g^−1^ FW), chlorophyll *ab* (905.21 μg g^−1^ FW), chlorophyll *b* (271.08 μg g^−1^ FW), β-cyanin (537.21 ng g^−1^ FW), betalain (1,121.93 ng g^−1^ FW), and carotenoids (105.08 mg 100 g^−1^) were recorded in *A. hypochondriacus*, which were fully corroborative to the findings of Khanam and Oba^43^ of green and red amaranth*.* Betalain, betacyanin, and betaxanthin content in the current investigation were much pronounced than betalain, betacyanin, and betaxanthin content of red morph amaranth^35^, green morph amaranth^37^, stem amaranth^38^, and weedy amaranth^36^, while the content of these color pigments of this study was corroborated with *A. blitum*^39^ of our earlier studies. Total carotenoids content in the current investigation was much greater than the total carotenoids content of green morph amaranth^37^, *A. spinosus* weedy amaranth^36^ and total carotenoids content of this study were corroborated with *A. viridis* weedy amaranth^36^, while total carotenoids content in the current investigation was lower than the total carotenoids content of red morph amaranth^35^, stem amaranth^38^ and *A. blitum*^39^ of our earlier studies.

### Bioactive components and radical scavenging capacity

The total polyphenols (TP), vitamin C, β-carotene, total antioxidant activity (TA), and total flavonoids (TF) contents of *A. hypochondriacus* are presented in Table 4. The highest content of β-carotene was recorded in the genotype AHC2 (82.34 mg 100 g^−1^), while the genotype AHC4 exhibited the lowest β-carotene content (48.33 mg 100 g^−1^ FW) with an average of 58.26 mg 100 g^−1^ FW. Five accessions had greater β-carotene contents than the mean value. The content of β-carotene was high in the genotypes AHC3, AHC5, and AHC8. The vitamin C exhibited pronounced variations regarding genotypes, which ranged from 11.97 mg 100 g^−1^ in the genotype AHC3 to 184.77 mg 100 g^−1^ in the genotype AHC11 with an average of 84.44 mg 100 g^−1^ FW. Six accessions had higher vitamin C contents than the grand mean value. High vitamin C content was recorded in the genotypes AHC1, AHC2, AHC9, and AHC4. The total polyphenols (TP) ranged from 13.23 GAE μg g^−1^ FW (AHC8) to 29.34 GAE μg g^−1^ FW (AHC11) with an average TP content of 24.04 GAE μg g^−1^ FW. High TP was noted in the genotypes AHC4, AHC2, AHC1, and AHC6. Six accessions exerted greater TP contents than the mean TP content. The pronounced variability was recorded in TF content regarding genotypes, which ranged from 106.80 RE μg g^−1^ DW ( AHC8) to 170.97 RE μg g^−1^ DW) ( AHC9). The average value of TF content was 147.26 RE μg g^−1^ DW. The highest TF content was recorded in the genotype AHC9 with the order of AHC9 ˃ AHC4 = AHC5 ˃ AHC10 = AHC1 ˃ AHC2 = AHC6. Six accessions had higher TF content than the mean TF content. The TA (DPPH) content was high and variability was not pronounced, which ranged from 16.27 Trolox equivalent antioxidant capacity (TEAC) μg g^−1^ DW (AHC8) to 33.46 TEAC μg g^−1^ DW (AHC6). The highest TA (DPPH) content was recorded in the genotypes AHC6, AHC4, AHC11, AHC5, and AHC10 (DPPH). Conversely, the lowest TA (DPPH) content was found in the genotype AHC8 with a mean TA (DPPH) value of 30.52 TEAC μg g^−1^ DW. Eight accessions exerted much greater TA (DPPH) contents than the mean TA (DPPH) content. Similar to TA (DPPH), TA (ABTS^+^) showed a similar trend, which validated the measurement of the quenching capacity of radicals in two methods. The TA (ABTS^+^) was high and variations were not pronounced, which ranged from 28.54 TEAC μg g^−1^ DW (AHC8) to 62.92 TEAC μg g^−1^ DW (AHC6). The highest TA (ABTS^+^) was noted in the genotypes AHC6, AHC4 AHC11, AHC5, and AHC10. Conversely, the lowest TA (ABTS^+^) was recorded in the genotype AHC8 with an average of 56.88 TEAC mg g^−1^ DW. Nine genotypes exhibited much higher TA (ABTS^+^) contents than the mean TA (ABTS^+^) value. We observed considerable vitamin C and β-carotene (184.77 and 82.34 mg 100 g^−1^) in the accessions of *A. hypochondriacus*, which were greater than the findings of red amaranth of our previous studies^10^*.* The TP (29.34 GAE μg g^−1^ FW) recorded in this investigation was much greater than the findings of green and red amaranth of Khanam et al.^44^. Our obtained TF (170.97 RE μg g^−1^ DW), TA (DPPH) (33.46 TEAC μg g^−1^ DW), and TA (ABTS^+^) (62.92 TEAC μg g^−1^ DW) in *A. hypochondriacus* were corroborated with the findings of red amaranth of Khanam et al.^44^, while the results of green amaranth of Khanam et al.^44^ had much lower values compared to our results*.* The beta-carotene obtained from *A. hypochondriacus* leafy vegetables was greater than our previous study of *A. spinosus* weedy amaranth^36^ and corroborated with beta-carotene of *A. viridis* weedy amaranth^36^, while it was lower than our previous study of red morph amaranth^35^, stem amaranth^38^, and *A. blitum*^39^. The ascorbic acid obtained from *A. hypochondriacus* leafy vegetables was greater than our previous studies of green morph amaranth^37^, *A. spinosus* weedy amaranth^36^, stem amaranth^38^ and lower than red morph amaranth^35^, *A. viridis* weedy amaranth^36^, and *A. blitum*^39^. TP content of the present study was greater than red morph amaranth^35^ and *A. spinosus* weedy amaranth^36^, while it showed lower results than *A. viridis* weedy amaranth^36^. Total flavonoids recorded in *A. hypochondriacus* leaves were greater than our previous studies of red morph amaranth^35^, green morph amaranth^37^ stem amaranth^38^ and *A. blitum*^39^, while TF content of *A. hypochondriacus* leaves was lower than of our earlier studies of weedy amaranth^36^. The antioxidant capacity in DPPH and antioxidant capacity in ABTS^+^ obtained from *A. hypochondriacus* leaves were greater than our previous study of red morph amaranth^35^, green morph amaranth^37^, weedy amaranth^36^, stem amaranth^38^, and *A. blitum*^39^. The genotypes AHC6, AHC4, AHC11, AHC5, and AHC10 had high phenolics, vitamins, pigments, and antioxidants including high TA. We can utilize these five genotypes as high-yielding varieties containing high antioxidant profiles. We could conclude that *A. hypochondriacus* leaves have abundant phenolics, pigments, flavonoids, vitamins, and antioxidants that offered enormous prospects for nourishing the vitamin and antioxidant scarce people and detail pharmacological study.

**Table 4:** Mean performance for vitamin C, TA (DPPH), TF, TP, and TA (ABTS^+^) of 11 *A. hypochondriacus* genotypes.

Genotypes	β-Carotene (mg 100 g^−1^ FW)	Vitamin C (mg 100 g^−1^ FW)	TP (GAE µg g^−1^ FW)	TF (RE µg g^−1^ DW)	TA (DPPH) (TEAC µg g^−1^ DW)	TA (ABTS^+^) (TEAC µg g^−1^ DW)
AHC1	68.06 ± 0.52c	135.58 ± 0.38b	26.86 ± 0.09c	152.55 ± 0.27c	30.61 ± 0.13d	57.22 ± 0.37d
AHC2	82.34 ± 0.57a	96.49 ± 0.48c	28.64 ± 0.09b	143.66 ± 0.32d	32.31 ± 0.13b	60.62 ± 0.18b
AHC3	78.82 ± 0.56b	11.97 ± 0.87i	21.52 ± 0.08f	135.34 ± 0.35e	31.58 ± 0.11c	59.16 ± 0.53c
AHC4	28.41 ± 0.56h	94.49 ± 0.82d	28.62 ± 0.12b	163.34 ± 0.29b	33.45 ± 0.12a	62.90 ± 0.31a
AHC5	68.46 ± 0.69c	72.01 ± 0.49f	19.69 ± 0.15g	163.41 ± 0.28b	33.24 ± 0.15a	62.48 ± 0.28a
AHC6	48.33 ± 0.62g	16.34 ± 0.19h	25.37 ± 0.07d	143.54 ± 0.36d	33.46 ± 0.09a	62.92 ± 0.41a
AHC7	57.82 ± 0.78e	66.63 ± 0.95g	21.35 ± 0.18f	133.71 ± 0.25e	25.61 ± 0.13g	47.22 ± 0.26g
AHC8	60.11 ± 0.75d	87.17 ± 0.26e	13.23 ± 0.15h	106.80 ± 0.29f	16.27 ± 0.07h	28.54 ± 0.46h
AHC9	51.68 ± 0.48f	97.70 ± 0.29c	23.87 ± 0.14e	170.97 ± 0.32a	32.65 ± 0.10b	61.30 ± 0.22b
AHC10	58.26 ± 0.72e	65.69 ± 0.38g	25.95 ± 0.14d	153.25 ± 0.36c	33.28 ± 0.10a	62.56 ± 0.33a
AHC11	57.64 ± 0.75e	184.77 ± 0.48a	29.34 ± 0.12a	153.25 ± 0.26c	33.31 ± 0.11a	60.72 ± 0.24a
Mean	58.26	84.44	24.04	147.26	30.52	56.88
Significance	**	**	**	**	**	**
CV%	1.51	2.23	3.76	1.51	1.12	0.88

*CV* coefficient of variation, *TA* total antioxidant capacity, *TP* total polyphenol content, *TF* total flavonoid content, n = 3; **Significant at 1% level.

### Analysis of correlation coefficient

The correlation of TA (DPPH), β-carotene, phytopigments, vitamin C, TF, TA (ABTS^+^), and TP of *A. hypochondriacus* are presented in Table 5. The above-mentioned correlation coefficients exhibited interesting results. All phytopigments exerted positive and significant interrelationships with TA (DPPH), TF, TA (ABTS^+^), and TP. It indicated that the increase in chlorophylls, carotenoids, β-xanthin, betalain, and β-cyanin content was directly associated with the increment of TA (ABTS^+^), TP, TA (DPPH), and TF. Similarly, vitamin C had positive and insignificant correlations with TA, TF, and TP, whereas it exerted negative and insignificant correlations with all phytopigments. In the earlier works of our research group in *A. tricolor*^9,11,12,16^; in red morph amaranth^35^, in green morph amaranth^37^ and Jiménez-Aguilar and Grusak^40^ in 15 amaranth species observed the similar results which were corroborative to our present study. TA (ABTS^+^), TF, TA (DPPH), TPC, and β-carotene showed a positive and significant correlation among each other. It indicated that TF, TP, and β-carotene had strong quenching capacity of radicals. Similarly, positive and significant associations versus TA (DPPH) and TA (ABTS^+^) ensured the confirmation of the quenching capacity of radicals of *A. hypochondriacus* by two methods. All TP, phytopigments, TF, and β-carotene had strong quenching capacity of radicals as these antioxidant phytochemicals showed significant correlations with TA (DPPH) and TA (ABTS^+^). In this study, we observed that TP, phytopigments, TF, and β-carotene of *A. hypochondriacus* had strong quenching capacity of radicals.

**Table 5:** The coefficient of correlation for phytopigments, TF, β-carotene, TA (DPPH), vitamin C, TP, and TA (ABTS^+^) of 11 *A. hypochondriacus* genotypes.

Traits	Chl *b* (µg g^−1^ FW)	Chl *ab* (µg g^−1^ FW)	β-cyanin (ng g^−1^ FW)	β-xanthin (ng g^−1^ FW)	Betalain (ng g^−1^ FW)	Carotenoids mg 100 g^−1^ FW)	β-carotene (mg 100 g^−1^ FW)	Vitamin C (mg 100 g^−1^ FW)	TP (GAE µg g^−1^ FW)	TF (RE µg g^−1^ DW)	TA (DPPH) (TEAC µg g^−1^ DW)	TA (ABTS^+^) (TEAC µg g^−1^ DW)
Chlorophyll *a* (µg g^−1^ FW)	0.95**	0.94**	0.88**	0.86**	0.94**	− 0.74**	− 0.62**	− 0.024	0.55**	0.68**	0.82**	0.78**
Chlorophyll *b* (µg g^−1^ FW)		0.86**	0.78**	0.77**	0.82**	− 0.72**	− 0.66**	− 0.021	0.67**	0.76**	0.84**	0.77**
Chlorophyll *ab* (µg g^−1^ FW)			0.77**	0.76*	0.66**	− 0.75**	− 0.66**	− 0.018	0.75**	0.72**	0.78**	0.76**
β-cyanin (ng g^−1^ FW)				0.92**	0.93**	− 0.82**	− 0.58**	− 0.132	0.62**	0.75**	0.88**	0.79**
β-xanthin (ng g^−1^ FW)					0.97**	− 0.77**	− 0.57**	− 0.123	0.77**	0.77**	0.84**	0.84**
Betalain (ng g^−1^ FW)						− 0.76**	− 0.69**	− 0.141	0.88**	0.74**	0.89**	0.87**
Carotenoids (mg 100 g^−1^ FW)							0.92**	− 0.181	0.79**	0.88**	0.87**	0.86**
β-Carotene (mg 100 g^−1^ FW)								− 0.242	0.53*	0.58**	0.74**	0.64**
Vitamin C (mg 100 g^−1^ FW)									0.23	0.14	0.15	0.02
TP (GAE µg g^−1^ FW)										0.86**	0.74**	0.94**
TF (RE µg g^−1^ DW)											0.78**	0.87**
TA (DPPH) (TEAC µg g^−1^ DW)												0.96**

*Chl a* chlorophyll *a,Chl ab* chlorophyll *ab, TA* total antioxidant capacity, *TP* total polyphenol content, *TF* total flavonoid content; **Significant at 1% level.

In conclusions, It is the first report on *A. hypochondriacus* leaves. We grew eleven *A. hypochondriacus* genotypes to evaluate nutraceuticals, bioactive components, and quenching capacity of radicals. *A. hypochondriacus* leaves contained ample iron, K, zinc, Ca, copper, Mg, manganese, TF, protein, TP, dietary fiber, phytopigments, β-carotene, TA, vitamin C, carbohydrates, and antioxidants. Thus we can make drinks from *A. hypochondriacus* containing plentiful nutraceuticals, flavonoids, β-carotene, phytopigments, phenolics, vitamin C, and antioxidants to combat hidden hunger and achieve nutritional and antioxidant sufficiency. Based on correlation coefficients, all bioactive compounds of *A. hypochondriacus* leaves exhibited strong antioxidant activity. Hence, *A. hypochondriacus* leaves with staple foods (e.g., rice, wheat, maize) could significantly contribute to reducing the occurrence of hidden hunger in the globe. The genotypes AHC6, AHC4, AHC11, AHC5, and AHC10 can be utilized for making drinks.

## Methods

### Experimental materials

Eleven *A. hypochondriacus* genotypes were evaluated in this experiment.

### Design and layout

We executed the experiment in three replicates following a completely randomized block design (RCBD) at Bangabandhu Sheikh Mujibur Rahman Agricultural University. Each genotype was grown in 1 m^2^ experimental plot following 20 cm and 5 cm distance between rows and plants, respectively.

### Intercultural practices

Recommended fertilizer doses, such as triple super phosphate, murate of potash, gypsum, and urea, at the rate of 100, 150, 30, and 200 kg/ha, respectively were applied^45^. Cultural practices were maintained appropriately^45^. Compost (10 ton/ha) was applied during preparation of lands^10^. For maintaining the exact spacing of plants in a row, proper thinning was executed. Weeds of experimental plots were regularly removed through proper weeding and hoeing. We provide regular irrigation in the experimental plots for maintaining the proper growth of vegetable amaranth. We collected the leaf samples at 30 days old plant.

### Solvent and reagents

Solvent: Acetone, hexane, and methanol. Reagents: dithiothreitol (DTT), cesium chloride, HClO_4_, HNO_3_, H_2_SO_4_, ascorbic acid, standard compounds of pure Trolox (6-hydroxy-2, 5, 7, 8-tetramethyl-chroman-2-carboxylic acid), Folin-Ciocalteu reagent, gallic acid, DPPH, rutin, ABTS^+^, 2, 2-dipyridyl, aluminum chloride hexahydrate, potassium acetate, sodium carbonate, and potassium persulfate.

### Estimation of proximate composition

AOAC method^22^ was followed to estimate the ash, moisture, crude fat, fiber, crude protein contents, and gross energy. The nitrogen was calculated following the Micro-Kjeldahl method. Finally, nitrogen was multiplied by 6.25 to measure crude protein (AOAC method 976.05). The total moisture, crude protein, ash, and crude fat (%) was subtracted from 100 for calculating carbohydrate (g kg^−1^ FW).

### Estimation of mineral composition

*A. hypochondriacus* leaf samples were dried in an oven at 70 °C for 24 h. Dried samples were ground in a mill. We determined calcium, potassium, magnesium, iron, manganese, copper, zinc, from powdered leaves following nitric-perchloric acid digestion method^22^. For this digestion, in the presence of carborundum beads 40 ml HClO_4_ (70%), 400 ml HNO_3_ (65%), and 10 ml H_2_SO_4_ (96%) were added to 0.5 g dried leaf sample. After digestion, the ascorbic acid method was followed to measure P through dilution of the solution appropriately in triplicate. We added ascorbic acid and antimony to the yellow-colored complex solution for converting it to a blue-colored phosphomolybdenum complex. Sarker and Oba^22^ method was followed to read the absorbance by atomic absorption spectrophotometry (AAS) (Hitachi, Tokyo, Japan) at wavelengths of 285.2 nm (magnesium), 76 6.5 nm (potassium), 248.3 nm (iron), 422.7 nm (calcium), 279.5 nm (manganese), 213.9 nm (zinc), 324.8 nm (copper).

### Determination of chlorophylls and carotenoids

Chlorophyll *ab*, chlorophyll *b*, carotenoids, and chlorophyll *a* were calculated by extracting the leaves in acetone (80%)^22,46^. A spectrophotometer (Hitachi, U-1800, Tokyo, Japan) was used to measure the absorbance at 646 nm for chlorophyll *b* and 663 nm for chlorophyll *a*, and 470 nm for carotenoids, respectively.

The formulae were given below:

Chlorophyll *a* (μg/mL) = C_*a*_ = 12.21 A_663_ − 2.81 A_646_.

Chlorophyll *b* (μg/mL) = C_*b*_ = 20.13 A_646_ − 5.03 A_663_.

Carotenoids (μg/mL) = (1000A_470_ – 3.27 C_*a*_ – 104 C_*b*_/229.

Where A_646_ = absorbance at a wavelength of 646 nm; A_663_ = absorbance at a wavelength of 663 nm; A_470_ = absorbance at a wavelength of 470 nm.

Finally, chlorophylls were calculated as micrograms per gram and carotenoids milligrams per 100 g of fresh weight, respectively.

### Betacyanins and betaxanthins content measurement

The leaves were extracted in 80% methyl alcohol containing 50 mM ascorbate to measure betacyanins and betaxanthins according to the method of Sarker and Oba^22^. A spectrophotometer (Hitachi, U-1800, Tokyo, Japan) was used to measure the absorbance at 540 nm for betacyanins and 475 nm for betaxanthins, respectively. The data were calculated as nanograms betanin equivalent per gram of fresh weight for betacyanins and nanograms indicaxanthin equivalent per gram of fresh weight for betaxanthins.

### Estimation of beta-carotene

For the estimation of beta-carotene, we followed our previously described method^22^. Exactly 500 mg of fresh leaf sample was added with 10 ml of 80% acetone and ground thoroughly in a mortar and pestle. The extract was centrifuged at 10,000 × *g* for 3–4 min. The final volume was marked up to 20 ml after removing the supernatant in a volumetric flask. A Hitachi (U-1800, Tokyo, Japan) spectrophotometer at 510 nm and 480 nm, respectively was set to take the absorbance. Data were expressed as milligrams beta-carotene per 100 g of fresh weight.

Beta-carotene = 7.6 (Abs. at 480) − 1.49 (Abs. at 510) × Final volume/ (1,000 × fresh weight of leaf).

### Estimation of ascorbic acid

A Hitachi spectrophotometer (U-1800, Tokyo, Japan) was utilized to estimate ascorbic acid (AsA) and dehydroascorbic acid (DHA) from the fresh leaves. Dithiothreitol (DTT) was used for the sample pre-incubation and reduction of dehydroascorbic acid into ascorbic acid. Ascorbic acid reduced ferric ion to ferrous ion. Reduced ferrous ion forms complexes with 2, 2-dipyridyl^22^. We read the absorbance of Fe^2+^ complexes with 2, 2-dipyridyl at 525 nm for estimation of vitamin C through the spectrophotometric (Hitachi, U-1800, Tokyo, Japan). We calculated vitamin C in milligrams per 100 g of fresh weight.

### Estimation of total polyphenols

Extraction of total polyphenols was carried out according to Jiménez-Aguilar and Grusak^40^ using 25 mg of sample in 2.5 mL of 1.2 M HCl containing methanol (90%) at 90 °C for 2 h in a water bath. With readjusting the volume (2.5 mL), the leaf extract was centrifuged at 7,500 rpm for 20 min. The leaf extracts (100 µL) were added to the Folin–Ciocalteau reagent (2 N, 50 µL). After 5 min, 2 N Na_2_CO_3_ (400 µL) and water (1 mL) was added. The leaf extracts were incubated for 90 min at 37 °C. Finally, it was removed to a microplate (flat bottom). In a microplate reader, the absorbance was detected at 740 nm. We estimated the results in equivalent to gallic acid (GAE) standard µg g^−1^ of FW. Note that results could be slightly overestimated because these samples contained ascorbic acid^47^.

### Estimation of total flavonoids

Total flavonoids were extracted and quantified according to the method described by Jiménez-Aguilar and Grusak^40^. Samples (100 mg) were mixed with 5 mL methanol (50%) in water and placed for 1 h with ultrasound. The leaf extracts were centrifuged for 10 min at 13,000 g (4 °C). The supernatants were then recovered. Flavonoid extracts (400 µL) were homogenized with water (500 µL), 5% NaNO_2_ (60 µL), 10% AlCl3 (140 µL). After 10 min, 1 mM NaOH (400 µL) was added. The leaf extracts were incubated for 10 min at a normal temperature. Finally, it was removed to a flat bottom microplate. The absorbance was read at 500 nm in a microplate reader. Results are expressed in µg of rutin equivalents (RE) per gram of sample DW.

### Radical quenching capacity assay

Thirty days old leaves were harvested. For the antioxidant capacity assay, the leaves were dried in the air in a shade. 40 ml aqueous methanol (90%) was utilized to extract grounded dried leaves (1 g) from each cultivar in a capped bottle (100 ml). A Thomastant T-N22S (Thomas Kagaku Co. Ltd., Japan) shaking water bath was utilized to extract leaf samples for 1 h. Exactly 0.45 µm filter (MILLEX-HV syringe filter, Millipore Corporation, Bedford, MA, USA) was used to filter the homogenized mixture. After centrifugation for 15 min at 10,000 × g, the antioxidant capacity was estimated from the filtered extract.

Diphenyl-picrylhydrazyl (DPPH) radical degradation method was used to estimate the antioxidant activity. We added 1 ml DPPH solution (250 µM) to 10 µl extract (in triplicate) in a test tube. After adding 4 ml distilled water the extract was placed in the dark for 30 min. A Hitachi U1800 spectrophotometer (Hitachi, Tokyo, Japan) was used to measure the absorbance at 517 nm. Method of Khanam et al.^44^ was followed for ABTS^+^ assay. To prepare two stock solutions separately ABTS^+^ solution of 7.4 mM and potassium persulfate of 2.6 mM were used. We mixed both solutions in equal proportion to prepare the working solution at room temperature. The working solution was allowed to react in the dark for 12 h. One hundred fifty μl extract was added to 2.85 ml of ABTS^+^ solution and allowed to react in the dark for 2 h. For the preparation of the solution, one ml of ABTS^+^ solution was mixed with sixty ml of methanol. A Hitachi spectrophotometer (U1800, Tokyo, Japan) was utilized to take the absorbance against methanol at 734 nm. The inhibition (%) of DPPH and ABTS^+^ corresponding with control was used to determine antioxidant capacity using the equation as follows:

Antioxidant activity (%) = (Abs. blank − Abs. sample/Abs. blank) × 100.

Where, Abs. blank is the absorbance of the control reaction [10 µl methanol for TAC (DPPH), 150 μl methanol for TAC (ABTS^+^) instead of leaf extract] and Abs. sample is the absorbance of the test compound. Trolox was used as the reference standard, and the results were expressed as μg Trolox equivalent g^−1^ DW.

### Statistical analysis

The **r**eplication mean was obtained by averaging the replication-wise sample data. We performed the analysis of variance (ANOVA) using Statistix 8 software. The mean separation was performed by Tukey’s HSD test at a probability level of 1%. We reported the results as the mean ± SD.

### Ethical statement

The lab and field experiment in this study was carried out following guidelines and recommendations of “Biosafety Guidelines of Bangladesh” published by the Ministry of Environment and Forest, Government of the People’s Republic of Bangladesh (2005).

## Data Availability

The data used in this manuscript will be available to the public.
